# The Impact of Perceived Discrimination on Cultural Identification, Psychological Stress, Emotion Regulation and Aggressive Tendencies in Individuals With Turkish Migration Background in Germany

**DOI:** 10.3389/fsoc.2022.705027

**Published:** 2022-06-16

**Authors:** Demet Dingoyan, Franka Metzner, Akin Kongur, Örsan Arslan, Gesa Elena Albertine Pust, Roland Weierstall-Pust

**Affiliations:** ^1^Institute of Medical Sociology, University Hospital Hamburg-Eppendorf, Hamburg, Germany; ^2^Institute of Medical Psychology, University Hospital Hamburg-Eppendorf, Hamburg, Germany; ^3^Educational Science With a Focus on Special Education (“Emotional and Social Development”), University of Siegen, Siegen, Germany; ^4^Department of Clinical Psychology and Psychotherapy, Medical School Hamburg, Hamburg, Germany; ^5^Department of Psychology, Medical School Hamburg, Hamburg, Germany; ^6^Oberberg Clinics Group, Berlin, Germany

**Keywords:** perceived discrimination, cultural identification, emotion regulation, aggressive tendencies, Turkish migration background

## Abstract

The following study considers correlates of the identification with the origin and host culture of German individuals with a Turkish migrant background. It examines how these two factors mediate the relationship between perceived discrimination, emotion regulation or psychological stress, and aggressive tendencies as the major dependent variable. For this purpose, the data of 229 people with Turkish migration background living in Germany was collected through an online survey. Findings depict that the identification with the Turkish (origin) and German (host) culture mediate the relationship between perceived discrimination and emotion regulation. The relationship between perceived discrimination and psychological stress is mediated by the identification with the German culture. The analysis shows that perceived discrimination is associated with a reduced identification with the German culture and with a high identification with the Turkish culture. Emotion regulation abilities are negatively related to perceived discrimination and identification with the Turkish culture. In contrary, the psychological stress level is positively related to perceived discrimination. The preparedness for aggressive behavior is also associated positively by psychological stress and negatively by emotion regulation abilities. The results are discussed against the background of the specific migration history and living conditions of Turkish immigrants in Germany.

## Introduction

Research focusing on acculturation and adjustment processes in a new cultural context after migration indicates a negative correlation between identification with the host culture and identification with the culture of origin (Hong et al., 2016). However, more recent studies suggest that the correlation between identification with the host culture and identification with the culture of origin can be positive as well as negative (Zander and Hannover, 2013; Hong et al., 2016). Furthermore, a meta-analysis including 83 studies showed a significant positive relationship between biculturalism and psychological-sociocultural adjustment, whereby this relationship was stronger than the relationship between having one dominant or heritage culture and adjustment (Nguyen and Benet-Martínez, 2013).

As some studies have shown, the development of identity through identification with a culture is particularly important during childhood and adolescence and can evolved in very different ways. A survey based on a sample with *n* = 5,000 adolescents from 26 various countries of origin and 13 host countries revealed a wide range of relationships, whereby positive associations between identification with the host culture and culture of origin were found particularly for immigration countries such as Canada, USA, Australia or New Zealand, and more negative associations for European countries (Berry et al., 2006). Berry et al. (2006) considered these differences to be due to an increased pressure to adjust and experiences of discrimination. In a longitudinal survey with two samples (*n* = 376 adolescents with Russian-German migration background in Germany; *n* = 549 individuals with Russian-Jewish migration background in Israel), a negative relationship between identification with the host culture and identification with the culture of origin was found for both samples. The higher the level of identification with the host culture, the more positive was the attitude toward the host culture, the language of the host country was spoken more often, and native adolescents were in their peer groups to a greater extent (Stoessel et al., 2012).

According to case studies on adolescents with a Turkish migration background in Germany, the cohesion between the culture of origin and the host culture can result in a dilemma, especially in the second generation. The adolescents described a kind of “sandwich position,” in which for example the same rather negative behavior is attributed as “typically Turkish” by teachers and as “typically German” by parents. This can lead to a misbalance between identification with two cultures, in which the identification with one culture dominates the other (Schmid, 2010).

It is assumed that this kind of unequal identity development leads to a limited repertoire of coping strategies as well as an increased vulnerability for stress, drug abuse, impairments of emotion control, and delinquent behavior (Stewart et al., 2000; Le and Stockdale, 2008; Yoo and Lee, 2008; Schmid, 2010). Individuals with a migration background who have had little success in these adjustment processes after migration showed numerous stress factors and loss of support through society or family (Espinosa et al., 2016).

In addition, experiences of racism and discrimination (e.g., unfair treatment in job interviews or at school, negative media reports) can also cause negative effects on mental health such as high blood pressure, increased heart rate, depression and anxiety symptoms (Espinosa et al., 2016) as well as problems in emotion control such as insults, attacks, or hidden micro-aggressions (Nadal, 2011; Yampolsky et al., 2016). The compensation of the resulting psychological strain (Schmidt et al., 2017) by trying to find a rebalance can initiate a suffering self-esteem and dysfunctional coping strategies. These can include impairments of emotion control or a self-concept that encourages criminal behavior (Yoo and Lee, 2008; Schmidt et al., 2017). According to Massarwi and Khoury-Kassabri (2017), the contradictory definition of one's own social identity in comparison to the host society can lead to the victimization of one's own cultural group. This can provoke feelings of being rejected or mistreated by the host society, as well as feelings of revenge and an increased tendency to violence.

Studies with people of African-American, Arab-American and Asian-American migration background have repeatedly shown a positive relationship between perceived discrimination and stress experience (Yampolsky et al., 2016). In a sample of *n* = 259 multicultural individuals, Yampolsky et al. (2016) found that experiences of discrimination and stress impaired the ability to combine different identities into the self-concept. In 204 Muslim individuals with a migration background in Italy, the negative connection between discrimination experiences and psychological well-being was more evident in the second generation of migrants than in the first generation (Giuliani et al., 2018).

The present study focused on discrimination experiences as the major independent variable due to its high significance in the discourse on mental health and aggressive tendencies in migrant populations (e.g., Mölsä et al., 2017; Straiton et al., 2019; Kira et al., 2019). The rejection-identification model postulated that negative consequences of racial discrimination on well-being can be slightly mitigated by identification with the minority group (Branscombe et al., 1999; Branscombe, 2002). Furthermore, according to the general stress theory, psychological and physical stress caused by racial discrimination can promote aggression-related conduct problems (Agnew, 1992, 2005; Simons et al., 2003, 2006). An analysis of the socio-economic panel showed that people individuals with a Turkish migration background in Germany feel discriminated more often than other migrant groups (Igel et al., 2010). von Lersner et al. (2015) found that adolescents with a Turkish migrant background in Germany were more exposed to psychological stress and stressors, e.g., during the acculturation process, than adolescents without a migration background (von Lersner et al., 2015).

Based on these theoretical frameworks and empirical findings, it was hypothesized that in people with a migration background experiences of discrimination can lead to a detachment from the culture of their new home country, and in turn impact their psychological health as well as increase stress-related aggressive tendencies. A sample of *n* = 229 individuals with a Turkish migration background living in Germany were interviewed by means of an online survey. According to the Federal Statistical Office (2019), individuals with a Turkish migration background are, with 3.4% of the German population, currently one of the largest groups amongst those with a migration background in Germany. Migration background means that individuals or their parents have a different citizenship from the German nationality, or they became naturalized (Federal Statistical Office, 2019). The aim of the study was to investigate the impact of (1) discrimination experiences as the major independent variable, (2) the identification with the German or Turkish culture, as well as the current psychological stress level and emotion regulation abilities as mediating variables, and (3) aggressive tendencies as the major outcome. To investigate this hypothesized relationship between the variables of interest, a path-model was calculated.

## Materials and Methods

### Subjects

For the present study, an a-priori sample size calculation was performed (Baltes-Götz, 2017) with a power (1 – β) 0.8 and an effect size of at least *f* = 0.05 for the full model including all variables of interest, using G^*^power 3.1 (Faul et al., 2009). A-priori sample size calculation is part of the research planning process and serves to identify the number of participants that is required to detect a given effect It is generally accepted as good practice in research (Kyriazos, 2018). A sample size of at least 209 participants was suggested. A total of 897 individuals accessed the survey. 597 (66.6%) did not start the survey after reading the general information. Incomplete data sets were removed to avoid the necessity to impute missing data. Overall, 229 full data sets were obtained (response rate = 25.5%). No systematic pattern could be identified *post hoc* for the 71 participants (7.9%) that dropped out and did not answer all items.

For the development of the online survey, the software UniPark (Questback, 2017) was used. Individuals with a Turkish migration background were sampled in the cities of Frankfurt/Main and Köln, Germany. The link to access the survey was distributed via local Internet platforms or advertisements in the local communities. Table 1 gives an overview on the socio-demographic characteristics of the sample.

**Table 1:** Socio-demographic data of the study sample (*N* = 229).

	***n***	**%**
**Gender**		
Female	122	53
Male	107	47
**Age group**		
18–20 years	11	5
21–30 years	113	49
31–40 years	47	21
41–50 years	27	12
>50 years	31	14
**Marital status**		
Single	106	46
Married	103	45
Cohabiting	17	7
Divorced	3	1
**Graduation**		
Certificate of Secondary Education (*Hauptschulabschluss*)	21	9
General Certificate of Secondary Education (*Realschulabschluss*)	51	22
University entrance diploma (*Abitur*)	40	18
Polytechnic degree/professional training (*Fachhochschulabschluss*/*Berufsausbildung*)	56	23
University degree (*Universitätsabschluss*)	44	19
No graduation/pupil	17	8
**Citizenship**		
German	99	43
Turkish	95	42
dual citizenship	35	15
**Generation status**		
1 (born in Turkey, immigration in adulthood)	27	12
1.25 (born in Turkey, immigration between the ages of 13 and 17)	32	14
1.5 (born in Turkey, immigration between the ages of 6 and 12)	32	14
1.75 (born in Turkey, immigration at pre-school age)	26	11
2 (born in Germany, both parents born in Turkey)	59	26
2.5 (born in Germany, one parent born in Germany)	26	11
3 (born in Germany, both parents born in Germany)	27	12

*Generation status refers to the classification defined by Rumbaut (1997)*.

Female and male individuals participated likewise. Most participants were in the age group from 21 to 30 years. Participants with a different educational background as well as different generation status participated. The number of participants with a German or Turkish citizenship was almost equally distributed.

### Procedures and Measures

To overcome language barriers, participants could choose between a German or a Turkish version of the survey. As validated questionnaires were only available in German, back-and-forth translations were used to translate the survey into Turkish, following common translation guidelines involving bilingual experts in clinical psychology (Gjersing et al., 2010). A total of 154 individuals (67%) completed the German version of the survey, 75 (33%) the Turkish version.

The study was carried out in compliance with the latest revision of the Declaration of Helsinki. All participants gave fully informed consent. After accessing the survey, general information on the study was provided first. The instruction provided (1) information on the study aims, (2) the estimated duration to complete the survey, and (3) a guarantee of complete anonymity and an emphasis on voluntary participation. After agreeing with the terms and conditions, the respondents were directed to the questionnaire.

A cross-sectional online survey was conducted. Firstly, participants were requested to report sociodemographic information and answer questions about their migration background and citizenship as well as their education background and relationship status. The immigrant generation was classified based on Rumbaut (1997) in the form of decimal generations, in which the age of the immigrant is also taken into account (Kemper, 2010). According to Rumbaut (1997), in addition to the classic generations of immigrants, there is the 1.75 generation (immigration at pre-school age), the 1.5 generation (immigration between age 6 and 12), the 1.25 generation (immigration between age 13 and 17), the 2.5 generation (only one of two parents was born in the host culture), and the generation 3 (both parents were born in the host culture). Secondly, questions measuring perceived discrimination, identification with German and Turkish culture, perceived psychological stress level, emotion regulation abilities and the preparedness to resort to aggressive behavior were presented. The following measures were administered. The instruments used in German and Turkish language can be requested from the authors.

#### Perceived Discrimination

To assess perceived discrimination, four items from the survey on perceived discrimination among young people with a Turkish ethnic background (Skrobanek, 2007) were selected, as well as two items from the “Fear of Discovery” scale (Jahnke et al., 2015). The items were selected to particularly cover experiences of discrimination related to the individual's cultural background. To select those items that particularly match discrimination experiences in this sample, the questionnaire and the appropriateness of each item had been carefully discussed with members from the respective community. For each item, the respondents had to indicate to which extent they agree with given statements, such as “*Due to my cultural background, I have experienced disadvantages in my school/at work/at my university*.” Responses were coded on a five-point scale ranging from “0” (*I totally disagree*) to “4” (*I totally agree*). To prove the validity of the newly composed items, an exploratory factor analysis was calculated (*principal axis factoring*). According to the Kaiser-Meyer-Olkin criterion of 0.73, the data was suitable for this type of analysis (Kaiser, 1958). According to the Scree-Plot, a single factor solution was preferable, with the main factor accounting for 53.0% of the scale variance. Cronbach's Alpha for the German version was 0.79 and 0.82 for the Turkish version of the survey, indicating acceptable scale reliability (Schecker, 2014).

#### Identification With the Turkish Culture

For the present study, the scale “Identification with the ancestry culture” (Maehler et al., 2008) was selected and rephrased to capture the identification with the Turkish culture. The scale on acculturation in Italian Canadians originates from research by Kim et al. (2001). Its German version has already successfully been used in German samples consisting of people with a migration background and has demonstrated its validity in the context of acculturation. Respondents have to indicate to which extent they agree with each of the five statements on identification regarding the respective culture, such as “*I'm proud of the Turkish culture*”. Reponses were coded on a five-point scale ranging from “0” (*I totally disagree*) to “4” (*I totally agree*). The five items are summed up for the calculation of a total score, ranging from 0 to 25 points. Higher values indicate a higher identification with the respective culture. The homogeneity of the scale measured by Cronbach's Alpha was satisfying for both, the German (0.94) and Turkish version (0.90) of the scale.

#### Identification With the German Culture

To assess the identification with the German culture, the scale “Identification with the ancestry culture” (Maehler et al., 2008) was selected and kept in its original version. For each item (for example “*I feel closely connected with German culture*”) responses were coded on a five-point scale ranging from “0” (*I totally disagree*) to “4” (*I totally agree*). The computation of the identification score was the same. For the German version, Cronbach's Alpha was 0.83 and 0.86 for the version translated into Turkish.

#### Psychological Stress

As a measure for the individual psychological stress level, the 25-item Sub-Clinical Stress Questionnaire (SSQ-25, Helms et al., 2016) was used. It consists of two sub-scales, covering psychological and physical stress signs. The SSQ-25 covers the most common stress signs defined in scientific literature and has demonstrated its usefulness for the application in non-clinical samples for the assessment of sub-clinical symptoms. For the present study, only the 15-item psychological stress sub-scale was considered. For each item (e.g., “*I found it hard to concentrate*”), the participant had to rate the intensity of the respective symptom in the past month on a five-point scale ranging from “0” (*not at all*) to “4” (*very strong*). The items were added up for the computation of a total score. The potential range of the total score was 0 to 60 points, with high values indicating a higher psychological stress level. Cronbach's Alpha in the present sample was 0.93 for the German version and 0.94 for the Turkish translation of the SSQ.

#### Emotion Regulation Abilities

To assess emotion regulation abilities, the emotion regulation scale from the questionnaire for the assessment of an individual's personal and social identity—short form (FPSI-K; Schmidt-Denter and Schick, 2005) was used. As for the other scales applied in this survey, the participant had to rate to which extent they agreed with the provided statements (for example “*Sometimes I can hardly fight against my mood*”) on a five-point scale ranging from “0” (*not at all*) to “4” (*very strong*). For the computation of the total score, the five item scores were added together. Higher values indicate better emotion regulation abilities. With a Crombach's Alpha of 0.89 for the German version and 0.81 for the Turkish translation of the survey, the homogeneity of the scale assessing emotion regulation abilities was satisfying.

#### Preparedness for Aggressive Behavior

Aggressive tendencies were assessed by ten items taken from the short questionnaire for assessing factors of aggression (K-FAF, Heubrock and Petermann, 2008). The K-FAF comprises 49 items in its original form and covers different facets of aggressive behavior. For the present study, the number of items has been reduced to ten in order to reduce the time needed to complete the questionnaire by ~20 min. Items that might be related to experiences of discrimination were selected (e.g., “*If someone provokes one of my friends, we avenge it together*”). An exploratory factor analysis was conducted to examine the structure for the German and for the Turkish version of the scale. For the German version, the structure of the scale was found to be suitable for factor analysis (KMO = 0.82) (Kaiser, 1958). Here, three factors had an eigenvalue >1 and were therefore greater than the Kaiser criterion. Based on the interpretation of the scree plot, a one-factor solution was chosen, which resolved 40.7% of the variance. For the Turkish version, the structure of the scale was found to be appropriate for factor analysis (KMO = 0. 7) (Kaiser, 1958). Here, three factors had an eigenvalue >1 and were therefore greater than the Kaiser criterion. Based on the interpretation of the scree plot, a one-factor solution was chosen, which resolved 40.3% of the variance. From the individual item scores, scored on the same five-point scale that ranged from “0” (*not at all*) to “4” (*very strong*), an overall score was calculated, with a range between 0 and 40 points. The reliability, estimated by Cronbach's Alpha, was satisfying, too (both versions = 0.83).

### Statistics

In a first step, zero-order Pearson correlations were calculated between the variables of interest. Calculations were performed using SPSS 26 for windows. The relations between experiences with discrimination on identification with the German or Turkish culture, the current psychological stress level as well as emotion regulation abilities and the preparedness to act aggressively were analyzed using path analyses in AMOS 26. To model the proposed mediated effect, the full model included (1) discrimination experiences as the independent variable, (2) the identification with the German or Turkish culture, as well as the current psychological stress level and emotion regulation abilities as intermediate variables, and (3) aggressive tendencies as the major dependent variable. Insignificant paths were deleted stepwise, using a backward elimination procedure. Assessment of overall model fit was based on multiple fit indices, including χ^2^, Root Mean Square Error of Approximation (RMSEA), Comparative Fit Index (CFI) and Normed Fit Index (NFI). An insignificant χ^2^ value between 1 and 2 is considered to be an adequate fit (Byrne, 2006). Samples with *n* ≤ 250 require a RMSEA value <0.08 (Hu and Bentler, 1999); CFI values approximating 0.95 and NFI values >0.90 are considered to represent a good model fit (Byrne, 1998; Hoyle, 2011).

## Result

### Zero-Order Correlations Between Perceived Discrimination, the Predictor Variables and Potential Confounding Variables

In a first step and before inclusion of the variables of interest into a path model, all zero-order correlations were determined (Table 2). Preparedness for aggressive behavior was associated with perceived discrimination, the psychological stress level and the emotion regulation abilities. There was a small negative correlation with the identification with the German culture, too.

**Table 2:** Zero-order correlations between Perceived Discrimination as well as the predictor variables and potential confounding variables.

**Variable**	***M ± SD* (range)**	**2**.	**3**.	**4**.	**5**.	**6**.
1. Perceived Discrimination	7.0 ± 5.2 (0–24)	*r* = 0.40 *p* < 0.001	*r* = −0.41 *p* < 0.001	*r* = 0.33 *p* < 0.001	*r* = −0.31 *p* < 0.001	*r* = 0.36 *p* < 0.001
2. Identification with the German culture	10.8 ± 5.3 (0–24)		*r* = −0.46 *p* < 0.001	*r* = 0.06 *p* = 0.397	*r* = 0.35 *p* < 0.001	*r* = −0.13 *p* = 0.044
3. Identification with the Turkish culture	16.7 ± 5.9 (0–24)			*r* = −0.03 *p* = 0.624	*r* = −0.32 *p* < 0.001	*r* = 0.07 *p* = 0.279
4. Psychological stress level	21.7 ± 12.8 (0–59)				*r* = −0.28 *p* < 0.001	*r* = 0.30 *p* < 0.001
5. Emotion regulation abilities	5.7 ± 3.0 (0–12)					*r* = −0.35 *p* < 0.001
6. Preparedness for aggressive behavior	11.4 ± 6.8 (0–40)					-

*r, Spearman correlation coefficient; p, level of significance*.

### Path Model for the Relation Between Perceived Discrimination, Identification With the Turkish or German Culture, the Psychological Stress Level, Emotion Regulation Abilities, and the Preparedness for Aggressive Behavior

**Figure 1:**
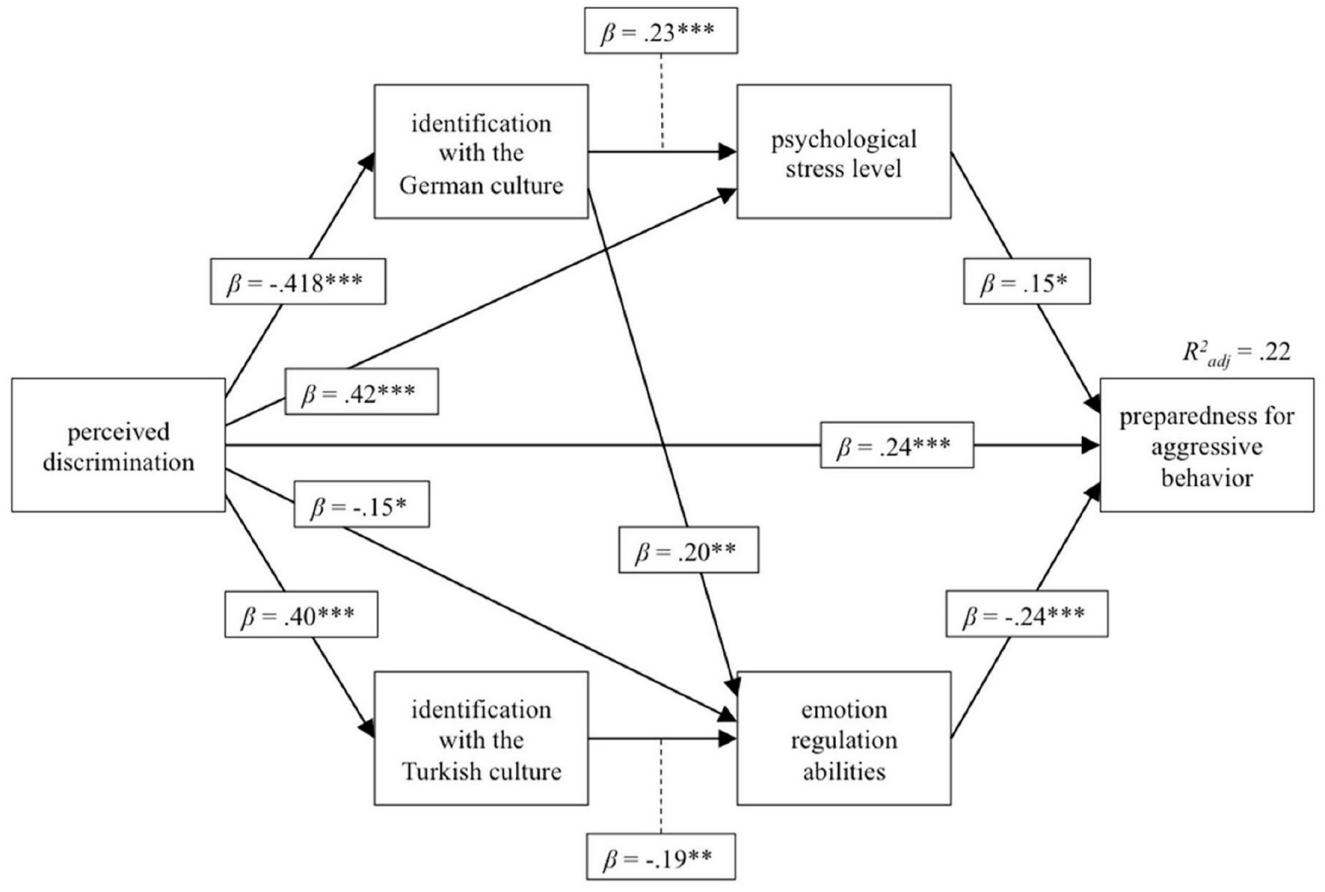
Path model for the relation between perceived discrimination, identification with the Turkish or German culture, the psychological stress level, emotion regulation abilities and the preparedness for aggressive behavior. ^*^P < 0.05, ^**^p < 0.01, ^***^p < 0.001; *R*^2^, explained variance; β_*s*_, standardized beta coefficient.

From the full model, insignificant paths were removed stepwise to optimize the model. The final model (*Chi*^2^ = 6.70, *p* = 0.082) is displayed in Figure 1. The indirect effect of perceived discrimination to the preparedness for aggressive behavior was calculated using Bayesian estimation. The experience of discrimination had an impact on all subsequent variables in the model and had the largest impact on the psychological stress level and the identification with the German culture as well as with the Turkish culture. In terms of mediation effects, those individuals who identified themselves more with the German culture had better emotion regulation abilities, but also higher psychological stress symptoms, whereas those who identified themselves more with the Turkish culture showed worse emotion regulation strategies. The preparedness to act aggressively was related to poor emotion regulation abilities, a higher psychological stress level and more experiences of discrimination. The three variables accounted for 22% of the variance. The additional indirect effect from the experiences of discrimination on the preparedness to behave aggressively was estimated *post-hoc*, using Bayesian estimation, and revealed another significant indirect impact of 0.12 (95% CI 0.03–0.25). Fit indices indicated a satisfying model fit (CFI = 0.99; NFI = 0.98, RMSEA = 0.07).

## Discussion

In the interviewed sample of individuals with a Turkish migration background living in Germany, the experiences of discrimination showed the largest significant impact on the (1) psychological stress level, (2) extent of the identification with the German culture and (3) extent of the identification with the Turkish culture. In particular, perceived discrimination was associated with a reduced identification with the German culture on the one hand and with a high identification with the Turkish culture on the other hand. The emotion abilities were negatively related to perceived discrimination and identification with the Turkish culture. In contrary, the psychological stress level was positively related to perceived discrimination. Furthermore, the preparedness for aggressive behavior was also associated positively by psychological stress and negatively by emotion regulation abilities.

The sample characteristics showed a balanced distribution of German citizenship (43%) and Turkish citizenship (42%). In contrast, identification with the Turkish culture was on average higher than identification with the German culture. Only 15% (*n* = 35) of participants stated that they had dual citizenship. However, a conclusion regarding the willingness to identify with both cultures (biculturalism) would not be valid, as dual citizenship in Germany for people with a Turkish migration background is only permitted in exceptional cases. At the time of the survey, the age distribution in the sample showed a high number of younger people (about 86% between 18 and 50 years old). Furthermore, about half of the participants were born in Germany (49%) and more often belonged to the second or third generation. About 39% reported that they migrated to Germany between early childhood and 17 years of age. In the line with the findings of Berry et al. (2006), it can be assumed that pressure to adjust and experiences of discrimination lead to a lower identification with the host culture, although the majority of the respondents had spent their childhood or youth in Germany.

In this context, it seems important to consider the migration history of Germany. On the basis of the German-Turkish recruitment agreement in 1961, the first generation of Turkish migrants predominantly came to Germany as labor workers (Gastarbeiter^*^innen) (Klopp, 2002). Another smaller group of Turkish migrants consisted of refugees, who fled to Germany due to political persecution (Sezer, 2001). For both groups, a long-term residence in Germany was initially not intended. So, there were little political efforts concerning the integration of the migrants into the host society. The main aim for both sides, the German government and the first-generation labor migrants, was to work in Germany for a limited time and then return to the country of origin (Butterwegge, 2005). Thus, they were exposed to many burdens, e.g., physically hard work and living in precarious conditions (Arin, 1981; Herbert, 1986). Some of the labor workers first left their children with relatives in Turkey and brought them to Germany when they were older (Karasu, 1981; Dzajic-Weber, 2016). Overall, it can be assumed that children of these labor migrants had to cope with specific burdens after migration, e.g., high adjustment requirements, partially ignored compulsory schooling, supporting their parents by taking care of younger siblings, and learning the German language (Akpinar, 1974; Klemm, 1979).

These historical developments and post-migration experiences may have far-reaching effects on the first-generation migrants themselves as well as on subsequent generations. Individuals with a Turkish migration background reported experiences of discrimination more frequently than other migrant groups in Germany (Igel et al., 2010; Morawa and Erim, 2014; Schunck et al., 2015; von Lersner et al., 2015). There is evidence that perceived discrimination and minority status negatively affect mental and physical health (Rapp et al., 2015; Schunck et al., 2015).

In a German interview sample (*n* = 653) it was found that people with a Turkish migration background were more likely to suffer from mental disorders at any time in life than the general population (Dingoyan et al., 2017). The findings on how the identification with a specific culture is associated with mental health among people with a migration background are limited and partly contradictory. For example, in the same study, female individuals with a Turkish migration background, who associated their cultural identity with the German culture, showed a risk for a depressive disorder that was about double as high in relation to individuals with a Turkish cultural identity (Janssen-Kallenberg et al., 2017). However, in a study conducted by Morawa et al. (2020) the acculturation style *separation*, which describes the retention of one's own culture without contact with the majority, was associated with higher levels of depressive symptoms in the first and second generation of study participants (*n* = 328) with a Turkish migration background than participants with other acculturation styles, whereas the second generation showed a higher severity of depressive symptoms. Earlier research shows that integration into the host society and a balance between identification with the culture of origin and the host culture can affect mental well-being and resilience to stress in a beneficial way (Haslam et al., 2005; Berry et al., 2006; Jones and Jetten, 2011; Ünlü Ince et al., 2014). In a study with adolescents with a Turkish migration background in Norway and Sweden identified predictors of good adaptation were Turkish identity, integration and a lower degree of perceived discrimination (Virta et al., 2004). Thus, identification with the host society can be seen as a prerequisite for integration, which encourages mental health. Considering the results of the present study, it can be assumed that due to failed integration efforts a balance between identification with the culture of origin and the host culture has been difficult to achieve. This presumably can lead to negative effects on the mental health of people with Turkish migration background and their descendants.

But, as the results of the present study also indicate, discrimination experiences can affect mental health and emotion regulation abilities in a negative way. Identification problems may occasionally just be a contributing factor, but not the sole reason for mental health problems, reduced emotion regulation abilities or preparedness for aggressive behavior. For example, appraisal and decision-making processes as well as biological and environmental factors can be assumed to be significant predictors for aggressive behavior, as described in the General Aggression Model by Allen et al. (2018). Discrimination experiences, stigmatization or social disadvantages which are closely associated with migration experiences, among other differentiating categories, can be assumed to be specific predictors for aggressive behavior (Huang et al., 2019). Further studies are needed to explain the identified associations without stereotyping.

When interpreting the results, limitations of the conducted study should be considered. The path model provided in the results section derives from cross-sectional data and does not show causality. Thus, the potential existence of reverse causality must be accounted for in the interpretation of the results. The authors of the present manuscript focused on perceived discrimination due to its high relevance for migrants' mental health. However, other potential causal relations could have been proposed: For example, it would also be possible that different cultural identifications lead to a different level of perceived discrimination or that perceived discrimination and cultural identification have a reciprocal relationship. Likewise, it could be suggested that dispositional aggressiveness would make participants more sensitive to stressful situations and less able to manage emotions, which in turn would be related to greater identification with the Turkish but not the German culture. This in turn would lead them to be more sensitive to any sign of discrimination and the perceived intensity. Moreover, the data was collected online. Meanwhile, even though online assessments are commonly used, there may still be a selection bias in the study sample, for example due to older people without possibility to access to the survey. Consequently, a sample of relatively young individuals was surveyed. It also cannot be ruled out, whether individuals with a certain opinion regarding the research question felt more attracted or less attracted to participate. Consequently, the results of the study are not necessarily generalizable to the population of people with a Turkish migration background in Germany. The results call for sub-group analyses to assess whether the identified associations depend on third variables, e.g., gender, migration generation or socioeconomic status. In future studies concerning the identification with the Turkish and German culture should not only be assessed dichotomously but should also consider biculturalism.

Nevertheless, the study results indicate that discrimination experiences and cultural identification might be important in order to understand and to address psychological stress and aggressive tendencies in people with migrant background. One starting point to strengthen the identification and satisfaction of migrants with the host country can be seen in the promotion of mutual trust (Leite et al., 2017).

## Data Availability Statement

The raw data supporting the conclusions of this article will be made available by the authors, without undue reservation.

## Ethics Statement

The studies involving human participants were reviewed and approved by Medical School Hamburg Ethics Committee. The patients/participants provided their written informed consent to participate in this study.

## Author Contributions

DD, FM, AK, ÖA, and RW-P designed the study. ÖA and AK were responsible for data collection. AK, GP, and RW-P conducted the statistical analysis and the data pre-processing. DD, FM, GP, and RW-P wrote the manuscript. All authors critically revised the manuscript, interpreted the results and gave critical feedback.

## Conflict of Interest

The authors declare that the research was conducted in the absence of any commercial or financial relationships that could be construed as a potential conflict of interest.

## Publisher's Note

All claims expressed in this article are solely those of the authors and do not necessarily represent those of their affiliated organizations, or those of the publisher, the editors and the reviewers. Any product that may be evaluated in this article, or claim that may be made by its manufacturer, is not guaranteed or endorsed by the publisher.
